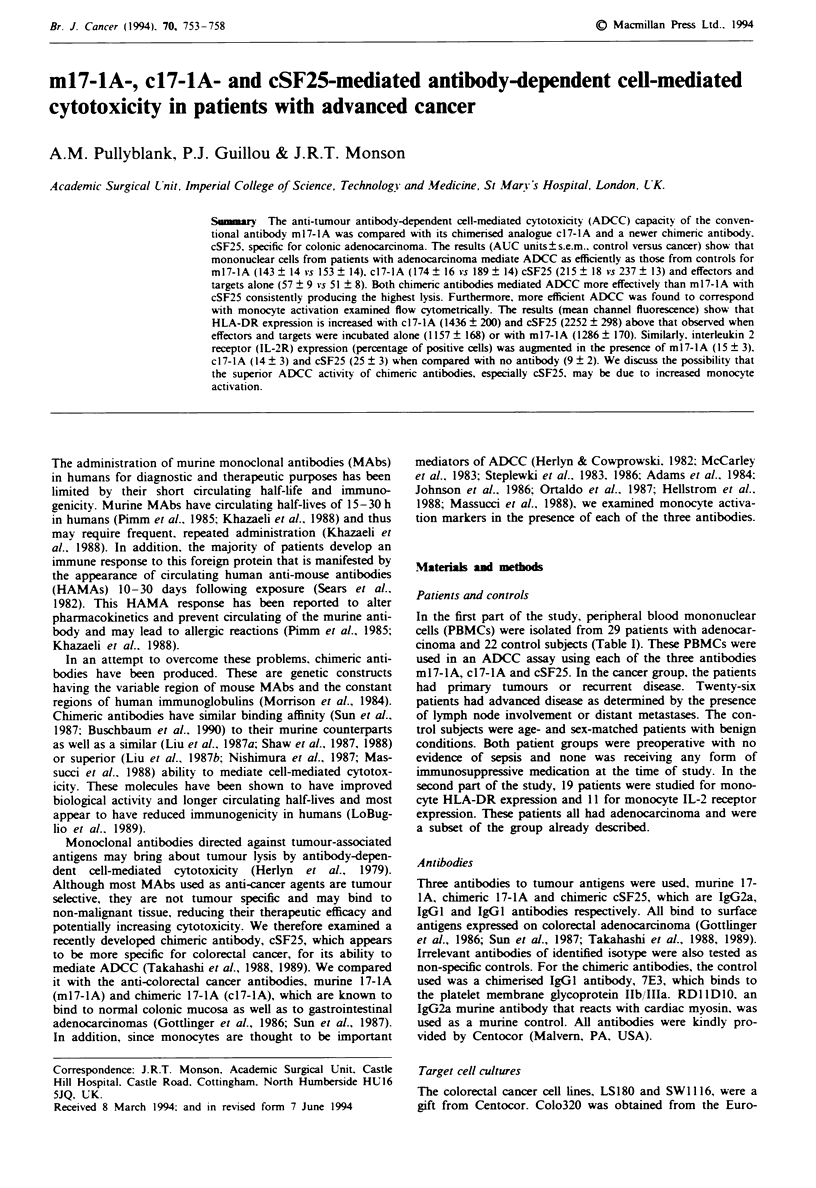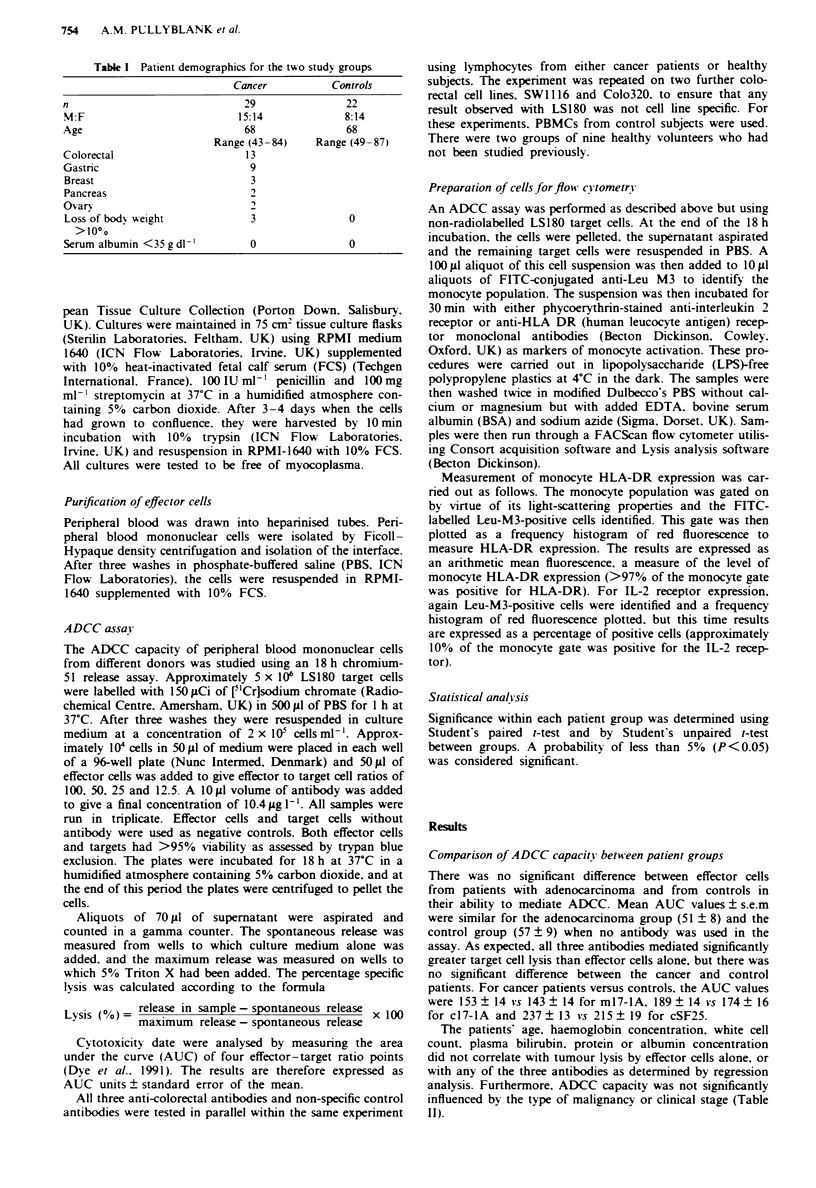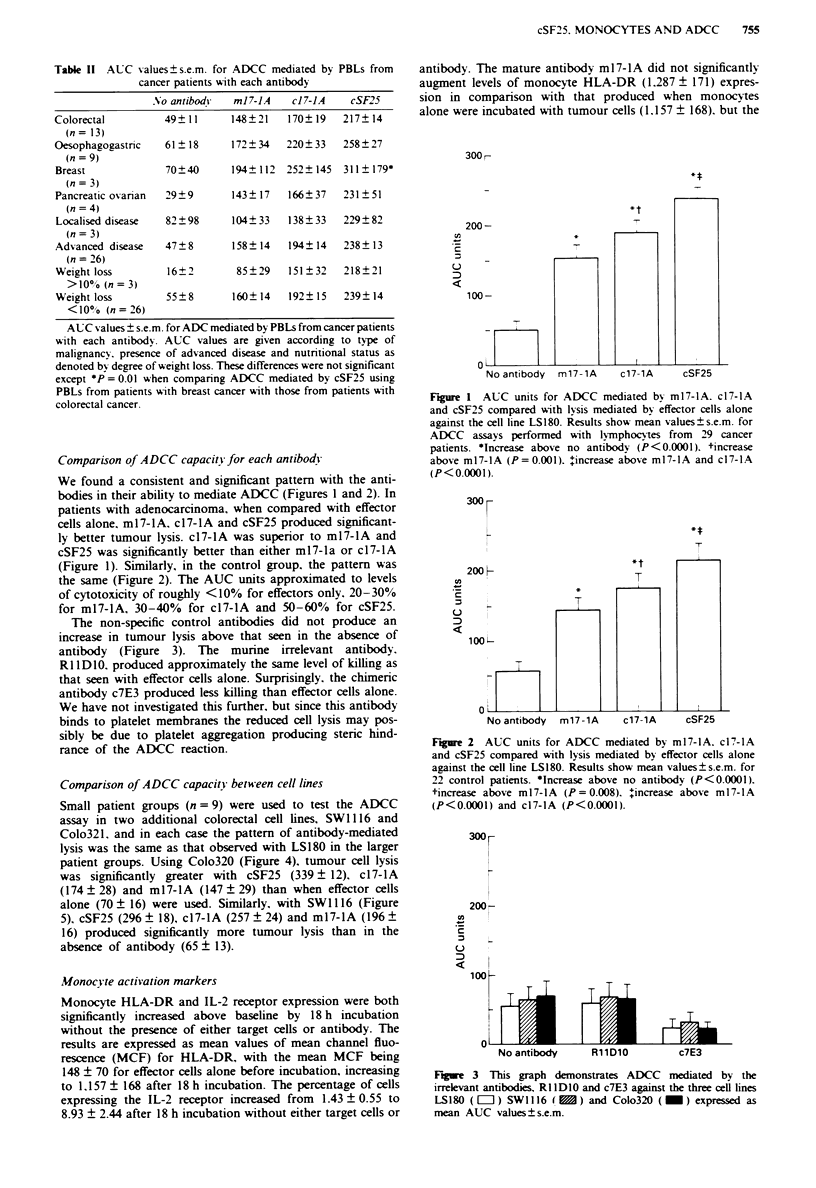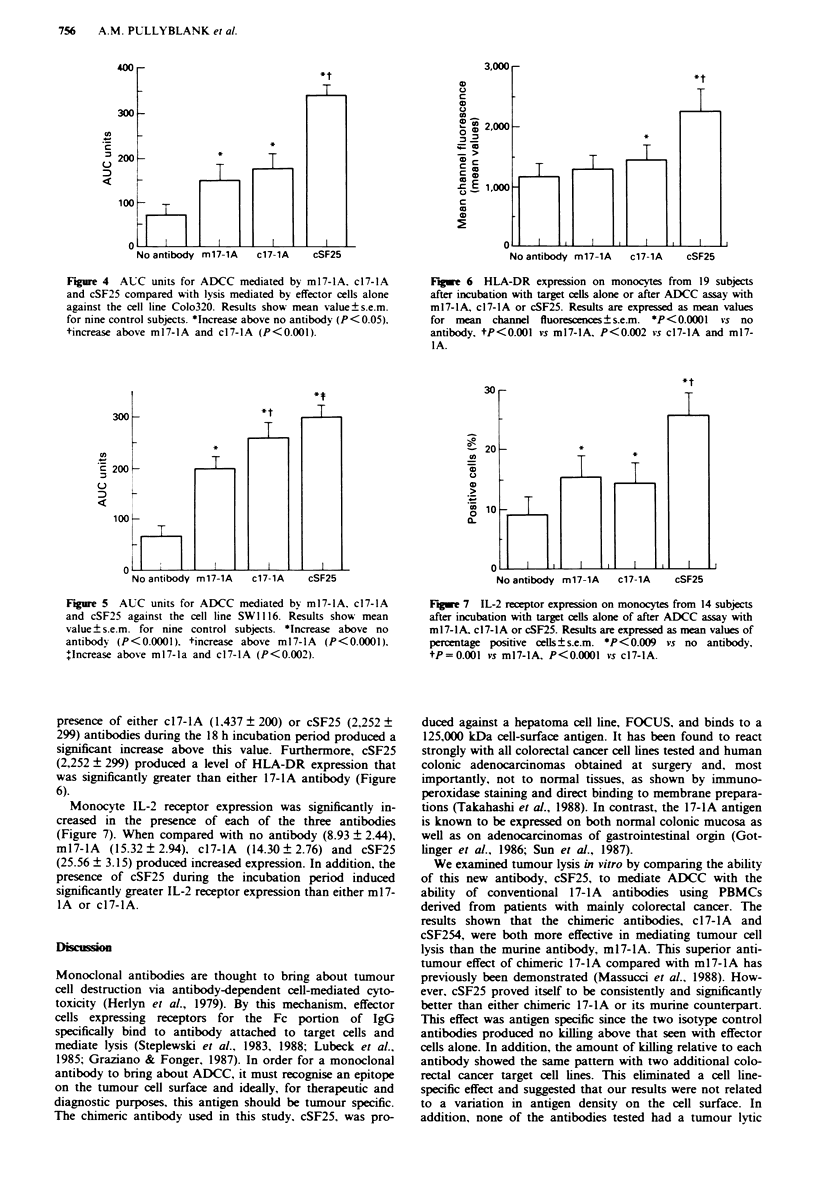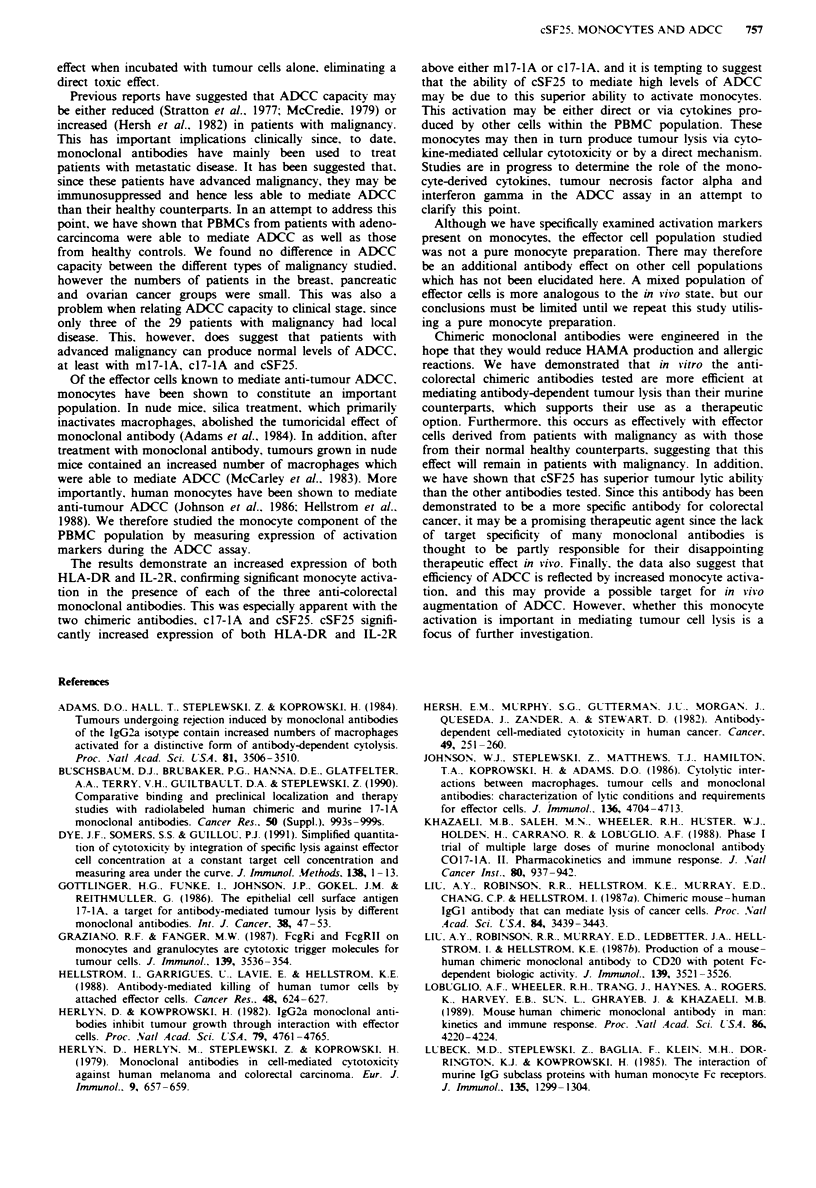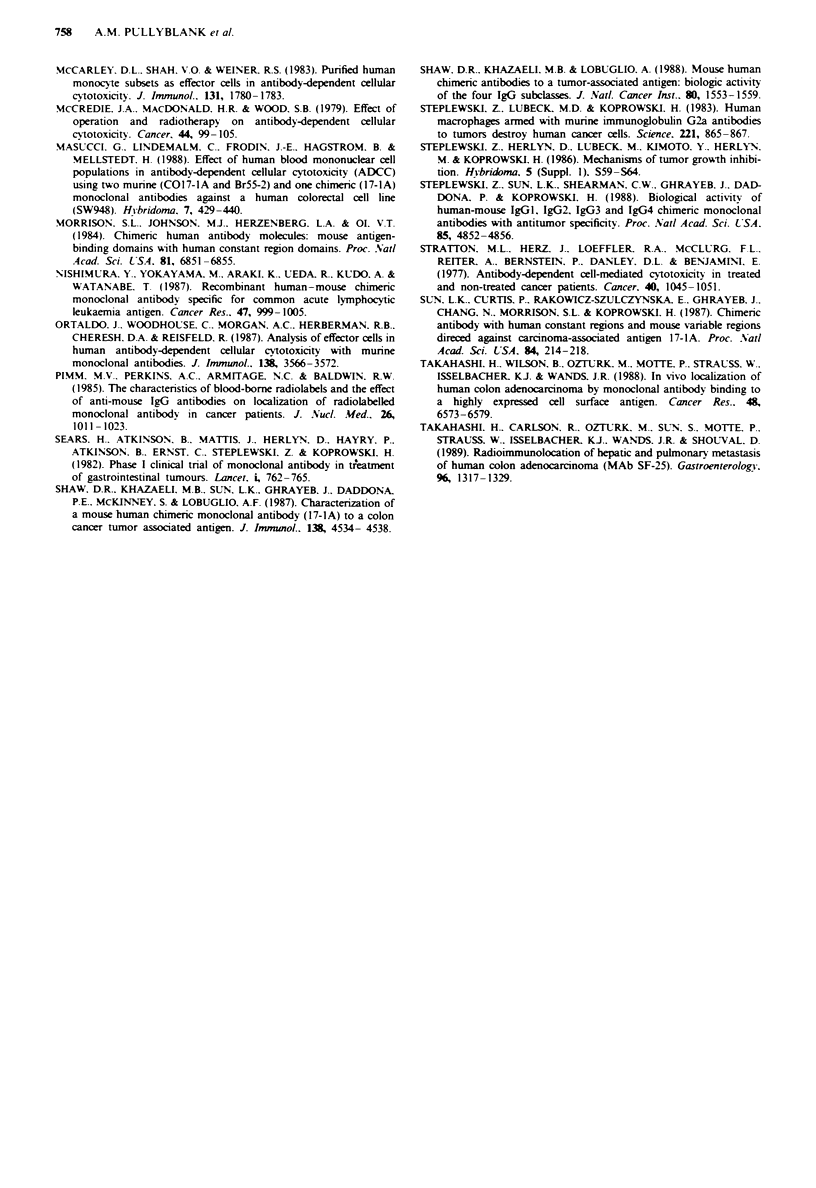# m17-1A-, c17-1A- and cSF25-mediated antibody-dependent cell-mediated cytotoxicity in patients with advanced cancer.

**DOI:** 10.1038/bjc.1994.390

**Published:** 1994-10

**Authors:** A. M. Pullyblank, P. J. Guillou, J. R. Monson

**Affiliations:** Academic Surgical Unit, Imperial College of Science, Technology and Medicine, St Mary's Hospital, London, UK.

## Abstract

The anti-tumour antibody-dependent cell-mediated cytotoxicity (ADCC) capacity of the conventional antibody m17-1A was compared with its chimerised analogue c17-1A and a newer chimeric antibody, cSF25, specific for colonic adenocarcinoma. The results (AUC units +/- s.e.m., control versus cancer) show that mononuclear cells from patients with adenocarcinoma mediate ADCC as efficiently as those from controls for m17-1A (143 +/- 14 vs 153 +/- 14), c17-1A (174 +/- 16 vs 189 +/- 14) cSF25 (215 +/- 18 vs 237 +/- 13) and effectors and targets alone (57 +/- 9 vs 51 +/- 8). Both chimeric antibodies mediated ADCC more effectively than m17-1A with cSF25 consistently producing the highest lysis. Furthermore, more efficient ADCC was found to correspond with monocyte activation examined flow cytometrically. The results (mean channel fluorescence) show that HLA-DR expression is increased with c17-1A (1436 +/- 200) and cSF25 (2252 +/- 298) above that observed when effectors and targets were incubated alone (1157 +/- 168) or with m17-1A (1286 +/- 170). Similarly, interleukin 2 receptor (IL-2R) expression (percentage of positive cells) was augmented in the presence of m17-1A (15 +/- 3), c17-1A (14 +/- 3) and cSF25 (25 +/- 3) when compared with no antibody (9 +/- 2). We discuss the possibility that the superior ADCC activity of chimeric antibodies, especially cSF25, may be due to increased monocyte activation.


Br. J. Cancer (1994), 70, 753-758 © Macmillan Press Ltd., 1994

### m17-1A-, c17-1A- and cSF25-mediated antibody-dependent cell-mediated cytotoxicity in patients with advanced cancer

A.M. Pullyblank, P.J. Guillou & J.R.T. Monson

*Academic Surgical Unit, Imperial College of Science, Technology and Medicine, St Mary's Hospital, London, UK.*

**Summary** The anti-tumour antibody-dependent cell-mediated cytotoxicity (ADCC) capacity of the conventional antibody m17-1A was compared with its chimerised analogue c17-1A and a newer chimeric antibody, cSF25, specific for colonic adenocarcinoma. The results (AUC units ± s.e.m., control versus cancer) show that mononuclear cells from patients with adenocarcinoma mediate ADCC as efficiently as those from controls for m17-1A (143 ± 14 *vs* 153 ± 14), c17-1A (174 ± 16 *vs* 189 ± 14) cSF25 (215 ± 18 *vs* 237 ± 13) and effectors and targets alone (57 ± 9 *vs* 51 ± 8). Both chimeric antibodies mediated ADCC more effectively than m17-1A with cSF25 consistently producing the highest lysis. Furthermore, more efficient ADCC was found to correspond with monocyte activation examined flow cytometrically. The results (mean channel fluorescence) show that HLA-DR expression is increased with c17-1A (1436 ± 200) and cSF25 (2252 ± 298) above that observed when effectors and targets were incubated alone (1157 ± 168) or with m17-1A (1286 ± 170). Similarly, interleukin 2 receptor (IL-2R) expression (percentage of positive cells) was augmented in the presence of m17-1A (15 ± 3), c17-1A (14 ± 3) and cSF25 (25 ± 3) when compared with no antibody (9 ± 2). We discuss the possibility that the superior ADCC activity of chimeric antibodies, especially cSF25, may be due to increased monocyte activation.

The administration of murine monoclonal antibodies (MAbs) in humans for diagnostic and therapeutic purposes has been limited by their short circulating half-life and immunogenicity. Murine MAbs have circulating half-lives of 15-30 h in humans (Pimm *et al.*, 1985; Khazaeli *et al.*, 1988) and thus may require frequent, repeated administration (Khazaeli *et al.*, 1988). In addition, the majority of patients develop an immune response to this foreign protein that is manifested by the appearance of circulating human anti-mouse antibodies (HAMAs) 10-30 days following exposure (Sears *et al.*, 1982). This HAMA response has been reported to alter pharmacokinetics and prevent circulating of the murine antibody and may lead to allergic reactions (Pimm *et al.*, 1985; Khazaeli *et al.*, 1988).

In an attempt to overcome these problems, chimeric antibodies have been produced. These are genetic constructs having the variable region of mouse MAbs and the constant regions of human immunoglobulins (Morrison *et al.*, 1984). Chimeric antibodies have similar binding affinity (Sun *et al.*, 1987; Buschbaum *et al.*, 1990) to their murine counterparts as well as a similar (Liu *et al.*, 1987*a*; Shaw *et al.*, 1987, 1988) or superior (Liu *et al.*, 1987*b*; Nishimura *et al.*, 1987; Massucci *et al.*, 1988) ability to mediate cell-mediated cytotoxicity. These molecules have been shown to have improved biological activity and longer circulating half-lives and most appear to have reduced immunogenicity in humans (LoBuglio *et al.*, 1989).

Monoclonal antibodies directed against tumour-associated antigens may bring about tumour lysis by antibody-dependent cell-mediated cytotoxicity (Herlyn *et al.*, 1979). Although most MAbs used as anti-cancer agents are tumour selective, they are not tumour specific and may bind to non-malignant tissue, reducing their therapeutic efficacy and potentially increasing cytotoxicity. We therefore examined a recently developed chimeric antibody, cSF25, which appears to be more specific for colorectal cancer, for its ability to mediate ADCC (Takahashi *et al.*, 1988, 1989). We compared it with the anti-colorectal cancer antibodies, murine 17-1A (m17-1A) and chimeric 17-1A (c17-1A), which are known to bind to normal colonic mucosa as well as to gastrointestinal adenocarcinomas (Gottlinger *et al.*, 1986; Sun *et al.*, 1987). In addition, since monocytes are thought to be important mediators of ADCC (Herlyn & Cowprowski, 1982; McCarley *et al.*, 1983; Steplewki *et al.*, 1983, 1986; Adams *et al.*, 1984; Johnson *et al.*, 1986; Ortaldo *et al.*, 1987; Hellstrom *et al.*, 1988; Massucci *et al.*, 1988), we examined monocyte activation markers in the presence of each of the three antibodies.

### Materials and methods

### *Patients and controls*

In the first part of the study, peripheral blood mononuclear cells (PBMCs) were isolated from 29 patients with adenocarcinoma and 22 control subjects (Table I). These PBMCs were used in an ADCC assay using each of the three antibodies m17-1A, c17-1A and cSF25. In the cancer group, the patients had primary tumours or recurrent disease. Twenty-six patients had advanced disease as determined by the presence of lymph node involvement or distant metastases. The control subjects were age- and sex-matched patients with benign conditions. Both patient groups were preoperative with no evidence of sepsis and none was receiving any form of immunosuppressive medication at the time of study. In the second part of the study, 19 patients were studied for monocyte HLA-DR expression and 11 for monocyte IL-2 receptor expression. These patients all had adenocarcinoma and were a subset of the group already described.

### *Antibodies*

Three antibodies to tumour antigens were used, murine 17-1A, chimeric 17-1A and chimeric cSF25, which are IgG2a, IgG1 and IgG1 antibodies respectively. All bind to surface antigens expressed on colorectal adenocarcinoma (Gottlinger *et al.*, 1986; Sun *et al.*, 1987; Takahashi *et al.*, 1988, 1989). Irrelevant antibodies of identified isotype were also tested as non-specific controls. For the chimeric antibodies, the control used was a chimerised IgG1 antibody, 7E3, which binds to the platelet membrane glycoprotein IIb/IIIa. RD11D10, an IgG2a murine antibody that reacts with cardiac myosin, was used as a murine control. All antibodies were kindly provided by Centocor (Malvern, PA, USA).

### *Target cell cultures*

The colorectal cancer cell lines, LS180 and SW1116, were a gift from Centocor. Colo320 was obtained from the Euro-

Correspondence: J.R.T. Monson, Academic Surgical Unit, Castle Hill Hospital, Castle Road, Cottingham, North Humberside HU16 5JQ, UK.
Received 8 March 1994; and in revised form 7 June 1994

Table I Patient demographics for the two study groups

| | Cancer | Controls |
|---|---|---|
| n | 29 | 22 |
| M:F | 15:14 | 8:14 |
| Age | 68<br>Range (43–84) | 68<br>Range (49–87) |
| Colorectal | 13 | |
| Gastric | 9 | |
| Breast | 3 | |
| Pancreas | 2 | |
| Ovary | 2 | |
| Loss of body weight >10% | 3 | 0 |
| Serum albumin <35 g dl$^{-1}$ | 0 | 0 |

pean Tissue Culture Collection (Porton Down, Salisbury, UK). Cultures were maintained in 75 cm$^2$ tissue culture flasks (Sterilin Laboratories, Feltham, UK) using RPMI medium 1640 (ICN Flow Laboratories, Irvine, UK) supplemented with 10% heat-inactivated fetal calf serum (FCS) (Techgen International, France), 100 IU ml$^{-1}$ penicillin and 100 mg ml$^{-1}$ streptomycin at 37°C in a humidified atmosphere containing 5% carbon dioxide. After 3–4 days when the cells had grown to confluence, they were harvested by 10 min incubation with 10% trypsin (ICN Flow Laboratories, Irvine, UK) and resuspension in RPMI-1640 with 10% FCS. All cultures were tested to be free of myocoplasma.

*Purification of effector cells*

Peripheral blood was drawn into heparinised tubes. Peripheral blood mononuclear cells were isolated by Ficoll–Hypaque density centrifugation and isolation of the interface. After three washes in phosphate-buffered saline (PBS, ICN Flow Laboratories), the cells were resuspended in RPMI-1640 supplemented with 10% FCS.

*ADCC assay*

The ADCC capacity of peripheral blood mononuclear cells from different donors was studied using an 18 h chromium-51 release assay. Approximately $5 \times 10^6$ LS180 target cells were labelled with 150 μCi of [$^{51}$Cr]sodium chromate (Radiochemical Centre, Amersham, UK) in 500 μl of PBS for 1 h at 37°C. After three washes they were resuspended in culture medium at a concentration of $2 \times 10^5$ cells ml$^{-1}$. Approximately $10^4$ cells in 50 μl of medium were placed in each well of a 96-well plate (Nunc Intermed, Denmark) and 50 μl of effector cells was added to give effector to target cell ratios of 100, 50, 25 and 12.5. A 10 μl volume of antibody was added to give a final concentration of 10.4 μg l$^{-1}$. All samples were run in triplicate. Effector cells and target cells without antibody were used as negative controls. Both effector cells and targets had >95% viability as assessed by trypan blue exclusion. The plates were incubated for 18 h at 37°C in a humidified atmosphere containing 5% carbon dioxide, and at the end of this period the plates were centrifuged to pellet the cells.

Aliquots of 70 μl of supernatant were aspirated and counted in a gamma counter. The spontaneous release was measured from wells to which culture medium alone was added, and the maximum release was measured on wells to which 5% Triton X had been added. The percentage specific lysis was calculated according to the formula

$$\text{Lysis }(\%) = \frac{\text{release in sample} - \text{spontaneous release}}{\text{maximum release} - \text{spontaneous release}} \times 100$$

Cytotoxicity date were analysed by measuring the area under the curve (AUC) of four effector–target ratio points (Dye *et al.*, 1991). The results are therefore expressed as AUC units ± standard error of the mean.

All three anti-colorectal antibodies and non-specific control antibodies were tested in parallel within the same experiment using lymphocytes from either cancer patients or healthy subjects. The experiment was repeated on two further colorectal cell lines, SW1116 and Colo320, to ensure that any result observed with LS180 was not cell line specific. For these experiments, PBMCs from control subjects were used. There were two groups of nine healthy volunteers who had not been studied previously.

*Preparation of cells for flow cytometry*

An ADCC assay was performed as described above but using non-radiolabelled LS180 target cells. At the end of the 18 h incubation, the cells were pelleted, the supernatant aspirated and the remaining target cells were resuspended in PBS. A 100 μl aliquot of this cell suspension was then added to 10 μl aliquots of FITC-conjugated anti-Leu M3 to identify the monocyte population. The suspension was then incubated for 30 min with either phycoerythrin-stained anti-interleukin 2 receptor or anti-HLA DR (human leucocyte antigen) receptor monoclonal antibodies (Becton Dickinson, Cowley, Oxford, UK) as markers of monocyte activation. These procedures were carried out in lipopolysaccharide (LPS)-free polypropylene plastics at 4°C in the dark. The samples were then washed twice in modified Dulbecco's PBS without calcium or magnesium but with added EDTA, bovine serum albumin (BSA) and sodium azide (Sigma, Dorset, UK). Samples were then run through a FACScan flow cytometer utilising Consort acquisition software and Lysis analysis software (Becton Dickinson).

Measurement of monocyte HLA-DR expression was carried out as follows. The monocyte population was gated on by virtue of its light-scattering properties and the FITC-labelled Leu-M3-positive cells identified. This gate was then plotted as a frequency histogram of red fluorescence to measure HLA-DR expression. The results are expressed as an arithmetic mean fluorescence, a measure of the level of monocyte HLA-DR expression (>97% of the monocyte gate was positive for HLA-DR). For IL-2 receptor expression, again Leu-M3-positive cells were identified and a frequency histogram of red fluorescence plotted, but this time results are expressed as a percentage of positive cells (approximately 10% of the monocyte gate was positive for the IL-2 receptor).

*Statistical analysis*

Significance within each patient group was determined using Student's paired *t*-test and by Student's unpaired *t*-test between groups. A probability of less than 5% ($P<0.05$) was considered significant.

### Results

*Comparison of ADCC capacity between patient groups*

There was no significant difference between effector cells from patients with adenocarcinoma and from controls in their ability to mediate ADCC. Mean AUC values ± s.e.m were similar for the adenocarcinoma group (51 ± 8) and the control group (57 ± 9) when no antibody was used in the assay. As expected, all three antibodies mediated significantly greater target cell lysis than effector cells alone, but there was no significant difference between the cancer and control patients. For cancer patients versus controls, the AUC values were 153 ± 14 *vs* 143 ± 14 for m17-1A, 189 ± 14 *vs* 174 ± 16 for c17-1A and 237 ± 13 *vs* 215 ± 19 for cSF25.

The patients' age, haemoglobin concentration, white cell count, plasma bilirubin, protein or albumin concentration did not correlate with tumour lysis by effector cells alone, or with any of the three antibodies as determined by regression analysis. Furthermore, ADCC capacity was not significantly influenced by the type of malignancy or clinical stage (Table II).

**Table II** AUC values ± s.e.m. for ADCC mediated by PBLs from cancer patients with each antibody

| | *No antibody* | *m17-1A* | *c17-1A* | *cSF25* |
|---|---|---|---|---|
| Colorectal ($n$ = 13) | 49 ± 11 | 148 ± 21 | 170 ± 19 | 217 ± 14 |
| Oesophagogastric ($n$ = 9) | 61 ± 18 | 172 ± 34 | 220 ± 33 | 258 ± 27 |
| Breast ($n$ = 3) | 70 ± 40 | 194 ± 112 | 252 ± 145 | 311 ± 179* |
| Pancreatic ovarian ($n$ = 4) | 29 ± 9 | 143 ± 17 | 166 ± 37 | 231 ± 51 |
| Localised disease ($n$ = 3) | 82 ± 98 | 104 ± 33 | 138 ± 33 | 229 ± 82 |
| Advanced disease ($n$ = 26) | 47 ± 8 | 158 ± 14 | 194 ± 14 | 238 ± 13 |
| Weight loss >10% ($n$ = 3) | 16 ± 2 | 85 ± 29 | 151 ± 32 | 218 ± 21 |
| Weight loss <10% ($n$ = 26) | 55 ± 8 | 160 ± 14 | 192 ± 15 | 239 ± 14 |

AUC values ± s.e.m. for ADC mediated by PBLs from cancer patients with each antibody. AUC values are given according to type of malignancy, presence of advanced disease and nutritional status as denoted by degree of weight loss. These differences were not significant except *$P$ = 0.01 when comparing ADCC mediated by cSF25 using PBLs from patients with breast cancer with those from patients with colorectal cancer.

*Comparison of ADCC capacity for each antibody*

We found a consistent and significant pattern with the antibodies in their ability to mediate ADCC (Figures 1 and 2). In patients with adenocarcinoma, when compared with effector cells alone, m17-1A, c17-1A and cSF25 produced significantly better tumour lysis. c17-1A was superior to m17-1A and cSF25 was significantly better than either m17-1a or c17-1A (Figure 1). Similarly, in the control group, the pattern was the same (Figure 2). The AUC units approximated to levels of cytotoxicity of roughly <10% for effectors only, 20–30% for m17-1A, 30–40% for c17-1A and 50–60% for cSF25.

The non-specific control antibodies did not produce an increase in tumour lysis above that seen in the absence of antibody (Figure 3). The murine irrelevant antibody, R11D10, produced approximately the same level of killing as that seen with effector cells alone. Surprisingly, the chimeric antibody c7E3 produced less killing than effector cells alone. We have not investigated this further, but since this antibody binds to platelet membranes the reduced cell lysis may possibly be due to platelet aggregation producing steric hindrance of the ADCC reaction.

*Comparison of ADCC capacity between cell lines*

Small patient groups ($n$ = 9) were used to test the ADCC assay in two additional colorectal cell lines, SW1116 and Colo321, and in each case the pattern of antibody-mediated lysis was the same as that observed with LS180 in the larger patient groups. Using Colo320 (Figure 4), tumour cell lysis was significantly greater with cSF25 (339 ± 12), c17-1A (174 ± 28) and m17-1A (147 ± 29) than when effector cells alone (70 ± 16) were used. Similarly, with SW1116 (Figure 5), cSF25 (296 ± 18), c17-1A (257 ± 24) and m17-1A (196 ± 16) produced significantly more tumour lysis than in the absence of antibody (65 ± 13).

*Monocyte activation markers*

Monocyte HLA-DR and IL-2 receptor expression were both significantly increased above baseline by 18 h incubation without the presence of either target cells or antibody. The results are expressed as mean values of mean channel fluorescence (MCF) for HLA-DR, with the mean MCF being 148 ± 70 for effector cells alone before incubation, increasing to 1,157 ± 168 after 18 h incubation. The percentage of cells expressing the IL-2 receptor increased from 1.43 ± 0.55 to 8.93 ± 2.44 after 18 h incubation without either target cells or antibody. The mature antibody m17-1A did not significantly augment levels of monocyte HLA-DR (1,287 ± 171) expression in comparison with that produced when monocytes alone were incubated with tumour cells (1,157 ± 168), but the

**Figure 1** AUC units for ADCC mediated by m17-1A, c17-1A and cSF25 compared with lysis mediated by effector cells alone against the cell line LS180. Results show mean values ± s.e.m. for ADCC assays performed with lymphocytes from 29 cancer patients. *Increase above no antibody ($P$ < 0.0001), †increase above m17-1A ($P$ = 0.001), ‡increase above m17-1A and c17-1A ($P$ < 0.0001).

**Figure 2** AUC units for ADCC mediated by m17-1A, c17-1A and cSF25 compared with lysis mediated by effector cells alone against the cell line LS180. Results show mean values ± s.e.m. for 22 control patients. *Increase above no antibody ($P$ < 0.0001), †increase above m17-1A ($P$ = 0.008), ‡increase above m17-1A ($P$ < 0.0001) and c17-1A ($P$ < 0.0001).

**Figure 3** This graph demonstrates ADCC mediated by the irrelevant antibodies, R11D10 and c7E3 against the three cell lines LS180 (□) SW1116 (▨) and Colo320 (■) expressed as mean AUC values ± s.e.m.

**Figure 4** AUC units for ADCC mediated by m17-1A, c17-1A and cSF25 compared with lysis mediated by effector cells alone against the cell line Colo320. Results show mean value±s.e.m. for nine control subjects. *Increase above no antibody ($P<0.05$). †increase above m17-1A and c17-1A ($P<0.001$).

**Figure 6** HLA-DR expression on monocytes from 19 subjects after incubation with target cells alone or after ADCC assay with m17-1A, c17-1A or cSF25. Results are expressed as mean values for mean channel fluorescences±s.e.m. *$P<0.0001$ *vs* no antibody, †$P<0.001$ *vs* m17-1A, $P<0.002$ *vs* c17-1A and m17-1A.

**Figure 5** AUC units for ADCC mediated by m17-1A, c17-1A and cSF25 against the cell line SW1116. Results show mean value±s.e.m. for nine control subjects. *Increase above no antibody ($P<0.0001$). †increase above m17-1A ($P<0.0001$). ‡Increase above m17-1a and c17-1A ($P<0.002$).

**Figure 7** IL-2 receptor expression on monocytes from 14 subjects after incubation with target cells alone of after ADCC assay with m17-1A, c17-1A or cSF25. Results are expressed as mean values of percentage positive cells±s.e.m. *$P<0.009$ *vs* no antibody, †$P=0.001$ *vs* m17-1A, $P<0.0001$ *vs* c17-1A.

presence of either c17-1A (1,437 ± 200) or cSF25 (2,252 ± 299) antibodies during the 18 h incubation period produced a significant increase above this value. Furthermore, cSF25 (2,252 ± 299) produced a level of HLA-DR expression that was significantly greater than either 17-1A antibody (Figure 6).

Monocyte IL-2 receptor expression was significantly increased in the presence of each of the three antibodies (Figure 7). When compared with no antibody (8.93 ± 2.44), m17-1A (15.32 ± 2.94), c17-1A (14.30 ± 2.76) and cSF25 (25.56 ± 3.15) produced increased expression. In addition, the presence of cSF25 during the incubation period induced significantly greater IL-2 receptor expression than either m17-1A or c17-1A.

### Discussion

Monoclonal antibodies are thought to bring about tumour cell destruction via antibody-dependent cell-mediated cytotoxicity (Herlyn *et al.*, 1979). By this mechanism, effector cells expressing receptors for the Fc portion of IgG specifically bind to antibody attached to target cells and mediate lysis (Steplewski *et al.*, 1983, 1988; Lubeck *et al.*, 1985; Graziano & Fonger, 1987). In order for a monoclonal antibody to bring about ADCC, it must recognise an epitope on the tumour cell surface and ideally, for therapeutic and diagnostic purposes, this antigen should be tumour specific. The chimeric antibody used in this study, cSF25, was produced against a hepatoma cell line, FOCUS, and binds to a 125,000 kDa cell-surface antigen. It has been found to react strongly with all colorectal cancer cell lines tested and human colonic adenocarcinomas obtained at surgery and, most importantly, not to normal tissues, as shown by immunoperoxidase staining and direct binding to membrane preparations (Takahashi *et al.*, 1988). In contrast, the 17-1A antigen is known to be expressed on both normal colonic mucosa as well as on adenocarcinomas of gastrointestinal orgin (Gotlinger *et al.*, 1986; Sun *et al.*, 1987).

We examined tumour lysis *in vitro* by comparing the ability of this new antibody, cSF25, to mediate ADCC with the ability of conventional 17-1A antibodies using PBMCs derived from patients with mainly colorectal cancer. The results shown that the chimeric antibodies, c17-1A and cSF254, were both more effective in mediating tumour cell lysis than the murine antibody, m17-1A. This superior anti-tumour effect of chimeric 17-1A compared with m17-1A has previously been demonstrated (Massucci *et al.*, 1988). However, cSF25 proved itself to be consistently and significantly better than either chimeric 17-1A or its murine counterpart. This effect was antigen specific since the two isotype control antibodies produced no killing above that seen with effector cells alone. In addition, the amount of killing relative to each antibody showed the same pattern with two additional colorectal cancer target cell lines. This eliminated a cell line-specific effect and suggested that our results were not related to a variation in antigen density on the cell surface. In addition, none of the antibodies tested had a tumour lytic effect when incubated with tumour cells alone, eliminating a direct toxic effect.

Previous reports have suggested that ADCC capacity may be either reduced (Stratton *et al.*, 1977; McCredie, 1979) or increased (Hersh *et al.*, 1982) in patients with malignancy. This has important implications clinically since, to date, monoclonal antibodies have mainly been used to treat patients with metastatic disease. It has been suggested that, since these patients have advanced malignancy, they may be immunosuppressed and hence less able to mediate ADCC than their healthy counterparts. In an attempt to address this point, we have shown that PBMCs from patients with adenocarcincoma were able to mediate ADCC as well as those from healthy controls. We found no difference in ADCC capacity between the different types of malignancy studied, however the numbers of patients in the breast, pancreatic and ovarian cancer groups were small. This was also a problem when relating ADCC capacity to clinical stage, since only three of the 29 patients with malignancy had local disease. This, however, does suggest that patients with advanced malignancy can produce normal levels of ADCC, at least with m17-1A, c17-1A and cSF25.

Of the effector cells known to mediate anti-tumour ADCC, monocytes have been shown to constitute an important population. In nude mice, silica treatment, which primarily inactivates macrophages, abolished the tumoricidal effect of monoclonal antibody (Adams *et al.*, 1984). In addition, after treatment with monoclonal antibody, tumours grown in nude mice contained an increased number of macrophages which were able to mediate ADCC (McCarley *et al.*, 1983). More importantly, human monocytes have been shown to mediate anti-tumour ADCC (Johnson *et al.*, 1986; Hellstrom *et al.*, 1988). We therefore studied the monocyte component of the PBMC population by measuring expression of activation markers during the ADCC assay.

The results demonstrate an increased expression of both HLA-DR and IL-2R, confirming significant monocyte activation in the presence of each of the three anti-colorectal monoclonal antibodies. This was especially apparent with the two chimeric antibodies, c17-1A and cSF25. cSF25 significantly increased expression of both HLA-DR and IL-2R above either m17-1A or c17-1A, and it is tempting to suggest that the ability of cSF25 to mediate high levels of ADCC may be due to this superior ability to activate monocytes. This activation may be either direct or via cytokines produced by other cells within the PBMC population. These monocytes may then in turn produce tumour lysis via cytokine-mediated cellular cytotoxicity or by a direct mechanism. Studies are in progress to determine the role of the monocyte-derived cytokines, tumour necrosis factor alpha and interferon gamma in the ADCC assay in an attempt to clarify this point.

Although we have specifically examined activation markers present on monocytes, the effector cell population studied was not a pure monocyte preparation. There may therefore be an additional antibody effect on other cell populations which has not been elucidated here. A mixed population of effector cells is more analogous to the *in vivo* state, but our conclusions must be limited until we repeat this study utilising a pure monocyte preparation.

Chimeric monoclonal antibodies were engineered in the hope that they would reduce HAMA production and allergic reactions. We have demonstrated that *in vitro* the anti-colorectal chimeric antibodies tested are more efficient at mediating antibody-dependent tumour lysis than their murine counterparts, which supports their use as a therapeutic option. Furthermore, this occurs as effectively with effector cells derived from patients with malignancy as with those from their normal healthy counterparts, suggesting that this effect will remain in patients with malignancy. In addition, we have shown that cSF25 has superior tumour lytic ability than the other antibodies tested. Since this antibody has been demonstrated to be a more specific antibody for colorectal cancer, it may be a promising therapeutic agent since the lack of target specificity of many monoclonal antibodies is thought to be partly responsible for their disappointing therapeutic effect *in vivo*. Finally, the data also suggest that efficiency of ADCC is reflected by increased monocyte activation, and this may provide a possible target for *in vivo* augmentation of ADCC. However, whether this monocyte activation is important in mediating tumour cell lysis is a focus of further investigation.

### References

ADAMS, D.O., HALL, T., STEPLEWSKI, Z. & KOPROWSKI, H. (1984). Tumours undergoing rejection induced by monoclonal antibodies of the IgG2a isotype contain increased numbers of macrophages activated for a distinctive form of antibody-dependent cytolysis. *Proc. Natl Acad. Sci. USA*, **81**, 3506-3510.

BUSCHSBAUM, D.J., BRUBAKER, P.G., HANNA, D.E., GLATFELTER, A.A., TERRY, V.H., GUILTBAULT, D.A. & STEPLEWSKI, Z. (1990). Comparative binding and preclinical localization and therapy studies with radiolabeled human chimeric and murine 17-1A monoclonal antibodies. *Cancer Res.*, **50** (Suppl.), 993s-999s.

DYE, J.F., SOMERS, S.S. & GUILLOU, P.J. (1991). Simplified quantitation of cytotoxicity by integration of specific lysis against effector cell concentration at a constant target cell concentration and measuring area under the curve. *J. Immunol. Methods*, **138**, 1-13.

GOTTLINGER, H.G., FUNKE, I., JOHNSON, J.P., GOKEL, J.M. & REITHMULLER, G. (1986). The epithelial cell surface antigen 17-1A, a target for antibody-mediated tumour lysis by different monoclonal antibodies. *Int. J. Cancer*, **38**, 47-53.

GRAZIANO, R.F. & FANGER, M.W. (1987). FcgRi and FcgRII on monocytes and granulocytes are cytotoxic trigger molecules for tumour cells. *J. Immunol.*, **139**, 3536-354.

HELLSTROM, I., GARRIGUES, U., LAVIE, E. & HELLSTROM, K.E. (1988). Antibody-mediated killing of human tumor cells by attached effector cells. *Cancer Res.*, **48**, 624-627.

HERLYN, D. & KOWPROWSKI, H. (1982). IgG2a monoclonal antibodies inhibit tumour growth through interaction with effector cells. *Proc. Natl Acad. Sci. USA*, **79**, 4761-4765.

HERLYN, D., HERLYN, M., STEPLEWSKI, Z. & KOPROWSKI, H. (1979). Monoclonal antibodies in cell-mediated cytotoxicity against human melanoma and colorectal carcinoma. *Eur. J. Immunol.*, **9**, 657-659.

HERSH, E.M., MURPHY, S.G., GUTTERMAN, J.U., MORGAN, J., QUESEDA, J., ZANDER, A. & STEWART, D. (1982). Antibody-dependent cell-mediated cytotoxicity in human cancer. *Cancer*, **49**, 251-260.

JOHNSON, W.J., STEPLEWSKI, Z., MATTHEWS, T.J., HAMILTON, T.A., KOPROWSKI, H. & ADAMS, D.O. (1986). Cytolytic interactions between macrophages, tumour cells and monoclonal antibodies: characterization of lytic conditions and requirements for effector cells. *J. Immunol.*, **136**, 4704-4713.

KHAZAELI, M.B., SALEH, M.N., WHEELER, R.H., HUSTER, W.J., HOLDEN, H., CARRANO, R. & LOBUGLIO, A.F. (1988). Phase I trial of multiple large doses of murine monoclonal antibody CO17-1A. II. Pharmacokinetics and immune response. *J. Natl Cancer Inst.*, **80**, 937-942.

LIU, A.Y., ROBINSON, R.R., HELLSTROM, K.E., MURRAY, E.D., CHANG, C.P. & HELLSTROM, I. (1987*a*). Chimeric mouse-human IgG1 antibody that can mediate lysis of cancer cells. *Proc. Natl Acad. Sci. USA*, **84**, 3439-3443.

LIU, A.Y., ROBINSON, R.R., MURRAY, E.D., LEDBETTER, J.A., HELLSTROM, I. & HELLSTROM, K.E. (1987*b*). Production of a mouse-human chimeric monoclonal antibody to CD20 with potent Fc-dependent biologic activity. *J. Immunol.*, **139**, 3521-3526.

LOBUGLIO, A.F., WHEELER, R.H., TRANG, J., HAYNES, A., ROGERS, K., HARVEY, E.B., SUN, L., GHRAYEB, J. & KHAZAELI, M.B. (1989). Mouse human chimeric monoclonal antibody in man: kinetics and immune response. *Proc. Natl Acad. Sci. USA*, **86**, 4220-4224.

LUBECK, M.D., STEPLEWSKI, Z., BAGLIA, F., KLEIN, M.H., DORRINGTON, K.J. & KOWPROWSKI, H. (1985). The interaction of murine IgG subclass proteins with human monocyte Fc receptors. *J. Immunol.*, **135**, 1299-1304.

MCCARLEY, D.L., SHAH, V.O. & WEINER, R.S. (1983). Purified human monocyte subsets as effector cells in antibody-dependent cellular cytotoxicity. *J. Immunol.*, **131**, 1780–1783.

MCCREDIE, J.A., MACDONALD, H.R. & WOOD, S.B. (1979). Effect of operation and radiotherapy on antibody-dependent cellular cytotoxicity. *Cancer*, **44**, 99–105.

MASUCCI, G., LINDEMALM, C., FRODIN, J.-E., HAGSTROM, B. & MELLSTEDT, H. (1988). Effect of human blood mononuclear cell populations in antibody-dependent cellular cytotoxicity (ADCC) using two murine (CO17-1A and Br55-2) and one chimeric (17-1A) monoclonal antibodies against a human colorectal cell line (SW948). *Hybridoma*, **7**, 429–440.

MORRISON, S.L., JOHNSON, M.J., HERZENBERG, L.A. & OI, V.T. (1984). Chimeric human antibody molecules: mouse antigen-binding domains with human constant region domains. *Proc. Natl Acad. Sci. USA*, **81**, 6851–6855.

NISHIMURA, Y., YOKAYAMA, M., ARAKI, K., UEDA, R., KUDO, A. & WATANABE, T. (1987). Recombinant human–mouse chimeric monoclonal antibody specific for common acute lymphocytic leukaemia antigen. *Cancer Res.*, **47**, 999–1005.

ORTALDO, J., WOODHOUSE, C., MORGAN, A.C., HERBERMAN, R.B., CHERESH, D.A. & REISFELD, R. (1987). Analysis of effector cells in human antibody-dependent cellular cytotoxicity with murine monoclonal antibodies. *J. Immunol.*, **138**, 3566–3572.

PIMM, M.V., PERKINS, A.C., ARMITAGE, N.C. & BALDWIN, R.W. (1985). The characteristics of blood-borne radiolabels and the effect of anti-mouse IgG antibodies on localization of radiolabelled monoclonal antibody in cancer patients. *J. Nucl. Med.*, **26**, 1011–1023.

SEARS, H., ATKINSON, B., MATTIS, J., HERLYN, D., HAYRY, P., ATKINSON, B., ERNST, C., STEPLEWSKI, Z. & KOPROWSKI, H. (1982). Phase I clinical trial of monoclonal antibody in treatment of gastrointestinal tumours. *Lancet*, **i**, 762–765.

SHAW, D.R., KHAZAELI, M.B., SUN, L.K., GHRAYEB, J., DADDONA, P.E., MCKINNEY, S. & LOBUGLIO, A.F. (1987). Characterization of a mouse human chimeric monoclonal antibody (17-1A) to a colon cancer tumor associated antigen. *J. Immunol.*, **138**, 4534– 4538.

SHAW, D.R., KHAZAELI, M.B. & LOBUGLIO, A. (1988). Mouse human chimeric antibodies to a tumor-associated antigen: biologic activity of the four IgG subclasses. *J. Natl. Cancer Inst.*, **80**, 1553–1559.

STEPLEWSKI, Z., LUBECK, M.D. & KOPROWSKI, H. (1983). Human macrophages armed with murine immunoglobulin G2a antibodies to tumors destroy human cancer cells. *Science*, **221**, 865–867.

STEPLEWSKI, Z., HERLYN, D., LUBECK, M., KIMOTO, Y., HERLYN, M. & KOPROWSKI, H. (1986). Mechanisms of tumor growth inhibition. *Hybridoma*, **5** (Suppl. 1), S59–S64.

STEPLEWSKI, Z., SUN, L.K., SHEARMAN, C.W., GHRAYEB, J., DADDONA, P. & KOPROWSKI, H. (1988). Biological activity of human-mouse IgG1, IgG2, IgG3 and IgG4 chimeric monoclonal antibodies with antitumor specificity. *Proc. Natl Acad. Sci. USA*, **85**, 4852–4856.

STRATTON, M.L., HERZ, J., LOEFFLER, R.A., MCCLURG, F.L., REITER, A., BERNSTEIN, P., DANLEY, D.L. & BENJAMINI, E. (1977). Antibody-dependent cell-mediated cytotoxicity in treated and non-treated cancer patients. *Cancer*, **40**, 1045–1051.

SUN, L.K., CURTIS, P., RAKOWICZ-SZULCZYNSKA, E., GHRAYEB, J., CHANG, N., MORRISON, S.L. & KOPROWSKI, H. (1987). Chimeric antibody with human constant regions and mouse variable regions direced against carcinoma-associated antigen 17-1A. *Proc. Natl Acad. Sci. USA*, **84**, 214–218.

TAKAHASHI, H., WILSON, B., OZTURK, M., MOTTE, P., STRAUSS, W., ISSELBACHER, K.J. & WANDS, J.R. (1988). In vivo localization of human colon adenocarcinoma by monoclonal antibody binding to a highly expressed cell surface antigen. *Cancer Res.*, **48**, 6573–6579.

TAKAHASHI, H., CARLSON, R., OZTURK, M., SUN, S., MOTTE, P., STRAUSS, W., ISSELBACHER, K.J., WANDS, J.R. & SHOUVAL, D. (1989). Radioimmunolocation of hepatic and pulmonary metastasis of human colon adenocarcinoma (MAb SF-25). *Gastroenterology*, **96**, 1317–1329.